# Supplementary material for: Smooth Interpolating Curves with Local Control and Monotone Alternating Curvature
Source: Comput Graph Forum. 2022 Oct 6;41(5):25–38. doi: 10.1111/cgf.14600 (PMC9827861; doi:10.1111/cgf.14600)
Supplement: Supplementary file 1 — Supplement Material [file CGF-41-25-s001.zip › Local-Smooth-Interpolating-MonoCurvature/extern/clothoids/submodules/Utils/docs/api-cpp/unabridged_orphan.html]

Full API — Utils v1.2

### Navigation

- index
- toc
- Utils »
- Full API

# Full API¶

## Directories¶

- Directory Utils
  - Files

## Files¶

- File Console.cc
  - Definition (`Console.cc`)
    - Program Listing for File Console.cc

- File Console.hxx
  - Definition (`Utils/Console.hxx`)
    - Program Listing for File Console.hxx

- File CPUinfo.cc
  - Definition (`CPUinfo.cc`)
    - Program Listing for File CPUinfo.cc

- File format.cc
  - Definition (`format.cc`)
    - Program Listing for File format.cc
  - Includes
  - Namespaces
  - Classes
  - Functions
  - Variables

- File Malloc.cc
  - Definition (`Malloc.cc`)
    - Program Listing for File Malloc.cc

- File Malloc.hxx
  - Definition (`Utils/Malloc.hxx`)
    - Program Listing for File Malloc.hxx

- File Numbers.cc
  - Definition (`Numbers.cc`)
    - Program Listing for File Numbers.cc

- File Numbers.hxx
  - Definition (`Utils/Numbers.hxx`)
    - Program Listing for File Numbers.hxx

- File os.cc
  - Definition (`os.cc`)
    - Program Listing for File os.cc
  - Includes
  - Defines

- File Quaternion.hxx
  - Definition (`Utils/Quaternion.hxx`)
    - Program Listing for File Quaternion.hxx

- File rang.cc
  - Definition (`rang.cc`)
    - Program Listing for File rang.cc

- File rang.hxx
  - Definition (`Utils/rang.hxx`)
    - Program Listing for File rang.hxx
  - Includes
  - Namespaces
  - Enums

- File Table.cc
  - Definition (`Table.cc`)
    - Program Listing for File Table.cc

- File Table.hxx
  - Definition (`Utils/Table.hxx`)
    - Program Listing for File Table.hxx

- File ThreadPool.hxx
  - Definition (`Utils/ThreadPool.hxx`)
    - Program Listing for File ThreadPool.hxx

- File TicToc.cc
  - Definition (`TicToc.cc`)
    - Program Listing for File TicToc.cc
  - Includes
  - Namespaces

- File TicToc.hxx
  - Definition (`Utils/TicToc.hxx`)
    - Program Listing for File TicToc.hxx

- File Token.cc
  - Definition (`Token.cc`)
    - Program Listing for File Token.cc

- File Token.hxx
  - Definition (`Utils/Token.hxx`)
    - Program Listing for File Token.hxx

- File Trace.cc
  - Definition (`Trace.cc`)
    - Program Listing for File Trace.cc

- File Trace.hxx
  - Definition (`Utils/Trace.hxx`)
    - Program Listing for File Trace.hxx

- File Utils.cc
  - Definition (`Utils.cc`)
    - Program Listing for File Utils.cc

- File Utils.hh
  - Definition (`Utils.hh`)
    - Program Listing for File Utils.hh
  - Included By

- File Utils.hxx
  - Definition (`Utils/Utils.hxx`)
    - Program Listing for File Utils.hxx

- File Utils\_CPU\_info.hh
  - Definition (`Utils_CPU_info.hh`)
    - Program Listing for File Utils\_CPU\_info.hh
  - Includes
  - Namespaces
  - Functions

- File Utils\_zstr.hh
  - Definition (`Utils_zstr.hh`)
    - Program Listing for File Utils\_zstr.hh

### Quick search

### Table of Contents

- rang - Colors for your Terminal
- Zstr
- C++ API

«
hide menu

menu
sidebar
»

### Navigation

- index
- toc
- Utils »
- Full API

© Copyright 2021, Enrico Bertolazzi.
Created using Sphinx 4.2.0.
